# Auditory spatial processing in Alzheimer’s disease

**DOI:** 10.1093/brain/awu337

**Published:** 2014-12-02

**Authors:** Hannah L. Golden, Jennifer M. Nicholas, Keir X. X. Yong, Laura E. Downey, Jonathan M. Schott, Catherine J. Mummery, Sebastian J. Crutch, Jason D. Warren

**Affiliations:** 1 Dementia Research Centre, UCL Institute of Neurology, University College London, London, WC1N 3BG, UK; 2 Department of Medical Statistics, London School of Hygiene and Tropical Medicine, London, WC1E 7HT, UK

**Keywords:** space, auditory, Alzheimer’s, posterior cortical atrophy, voxel-based morphometry

## Abstract

The location and motion of sounds in space are important cues for encoding the auditory world. Spatial processing is a core component of auditory scene analysis, a cognitively demanding function that is vulnerable in Alzheimer’s disease. Here we designed a novel neuropsychological battery based on a virtual space paradigm to assess auditory spatial processing in patient cohorts with clinically typical Alzheimer’s disease (*n* = 20) and its major variant syndrome, posterior cortical atrophy (*n* = 12) in relation to healthy older controls (*n* = 26). We assessed three dimensions of auditory spatial function: externalized versus non-externalized sound discrimination, moving versus stationary sound discrimination and stationary auditory spatial position discrimination, together with non-spatial auditory and visual spatial control tasks. Neuroanatomical correlates of auditory spatial processing were assessed using voxel-based morphometry. Relative to healthy older controls, both patient groups exhibited impairments in detection of auditory motion, and stationary sound position discrimination. The posterior cortical atrophy group showed greater impairment for auditory motion processing and the processing of a non-spatial control complex auditory property (timbre) than the typical Alzheimer’s disease group. Voxel-based morphometry in the patient cohort revealed grey matter correlates of auditory motion detection and spatial position discrimination in right inferior parietal cortex and precuneus, respectively. These findings delineate auditory spatial processing deficits in typical and posterior Alzheimer’s disease phenotypes that are related to posterior cortical regions involved in both syndromic variants and modulated by the syndromic profile of brain degeneration. Auditory spatial deficits contribute to impaired spatial awareness in Alzheimer’s disease and may constitute a novel perceptual model for probing brain network disintegration across the Alzheimer’s disease syndromic spectrum.

## Abstract (precis)

Auditory spatial processing is vulnerable in dementia. Golden *et al.* show that patients with typical Alzheimer’s disease or posterior cortical atrophy are impaired relative to controls in detecting the movement and location of sounds. The deficits have anatomical correlates in right parietal cortex, with implications for studies of network degeneration.

## Introduction

Sound is a major source of information from the world around us, particularly where vision is unavailable or reduced. Auditory scene analysis and localization of sounds in space entail formidable computational problems (Bregman, 1990): these are solved efficiently and automatically by the normal brain but potentially significant in brain disorders associated with reduced spatial acuity, such as Alzheimer’s disease. However, the clinical and neurobiological correlates of auditory spatial processing in Alzheimer’s disease have not been clarified.

Perception of sound location and movement typically demands precise integration of dynamic acoustic cues including inter-aural time and intensity differences and monaural pinna reflections (Blauert, 1997; Heller and Richards, 2010); such processing may be particularly vulnerable in Alzheimer’s disease. Patients with Alzheimer’s disease frequently complain of difficulty following a conversation in a busy room or over a noisy telephone line and to generic deficits of central auditory processing (Strouse *et al.*, 1995; Gates *et al.*, 1996, 2008, 2011; Golob *et al.*, 2007, 2009; Goll *et al.*, 2011). Deficits of auditory scene analysis have been demonstrated in Alzheimer’s disease (Goll *et al.*, 2012), as well as specific impairment in auditory spatial localization (Kurylo *et al.*, 1993). Functional neuroimaging and electrophysiological studies in the healthy human brain (Warren *et al.*, 2002; Zatorre *et al.*, 2002; Warren and Griffiths, 2003; Arnott *et al.*, 2004; Spierer *et al.*, 2008) have shown that auditory spatial information is preferentially processed by cortical mechanisms comprising a dorsally directed network including posterior superior temporal lobe and inferior parietal and prefrontal projection zones that are also key sites of involvement in Alzheimer’s disease (Warren *et al.*, 2012). Although Alzheimer’s disease is generally led by episodic memory impairment with supervening parietal and more widespread cognitive deficits (Perry and Hodges, 1999; Lambon Ralph *et al.*, 2003; Dubois *et al.*, 2007), variations on this typical syndrome of Alzheimer’s disease frequently occur. The most common and best characterized of these is the syndrome of posterior cortical atrophy (PCA), which is dominated by visual spatial or object deficits with relatively spared episodic memory (Galton *et al.*, 2000; Renner *et al.*, 2004; McMonagle *et al.*, 2006; Crutch *et al.*, 2012) in association with parietal and occipitotemporal hypometabolism and volume loss (Benson *et al.*, 1988; Tang-Wai *et al.*, 2004; Lehmann *et al.*, 2011). In large case series, underlying Alzheimer’s disease pathology has been found in most patients presenting with PCA (Tang-Wai *et al.*, 2004; Ikonomovic *et al.*, 2008; Crutch *et al.*, 2012). The nosological boundaries of typical Alzheimer’s disease, PCA and other variant Alzheimer’s disease syndromes and the extent to which these syndromes share pathophysiological and neuroanatomical substrates remain to be resolved (Warren *et al.*, 2012; Lehmann *et al.*, 2013). Investigation of non-canonical cognitive and behavioural functions is an important avenue for defining syndrome boundaries and commonalities across the Alzheimer’s disease spectrum (Hawkes, 2006; Warren *et al.*, 2012; Witoonpanich *et al.*, 2013): in this regard, auditory spatial processing is an attractive candidate function that engages relevant, distributed brain networks but harnesses a distinct sensory system complementary to the conventionally studied paradigm of vision (Bremmer *et al.*, 2001; Lewald *et al.*, 2002; Cohen, 2009; Salo *et al.*, 2013).

Besides dorsal temporo-parietal regions with an established role in spatial representation and analysis, spatial sound processing may engage additional brain regions, including retrosplenial cortex: activity in this region is modulated by on-line representation of auditory information, imagery, working memory and attention during auditory scene analysis (Pallesen *et al.*, 2009; Wong *et al.*, 2009; Zündorf *et al.*, 2013). Retrosplenial cortical areas (posterior cingulate and precuneus) are key components of a core temporo-parieto-frontal brain network that is likely to be integral to the pathogenesis of Alzheimer’s disease (Baron *et al.*, 2001; Frisoni *et al.*, 2002; Buckner *et al.*, 2005; Dickerson *et al.*, 2009; Seeley *et al.*, 2009; Lehmann *et al.*, 2010; Warren *et al.*, 2012). This so-called ‘default mode network’ shows correlated activity in the healthy ‘resting’ brain (Raichle *et al.*, 2001) and deactivates with certain tasks (Shulman *et al.*, 1997) but has also been implicated in various ‘active’ processes including maintenance of internal sensory representations (Buckner and Carroll, 2007; Buckner *et al.*, 2008; Spreng and Grady, 2010; Zvyagintsev *et al.*, 2013). More directly, auditory spatial as well as other aspects of auditory scene analysis have been shown to depend on retrosplenial cortex in healthy individuals (Wong *et al.*, 2009; Zündorf *et al.*, 2013) and in patients with Alzheimer’s disease (Goll *et al.*, 2012). Collectively, this evidence suggests auditory spatial processing may be an informative paradigm for understanding clinical symptoms and for probing brain network dysfunction in Alzheimer’s disease.

Here we undertook a systematic cognitive and neuroanatomical analysis of auditory spatial processing in typical Alzheimer’s disease and PCA. We designed a novel neuropsychological battery to interrogate different aspects of auditory space analysis, based on virtual acoustic space techniques: using these techniques, percepts of sounds at fixed locations or moving outside the head are created by simulating digitally the filtering effects of the pinnae (Wightman and Kistler, 1989*a*, *b*). Such techniques enable acoustic space parameters to be specified precisely and allow auditory spatial stimuli to be delivered conveniently and uniformly via headphones. We compared performance of patient cohorts with typical Alzheimer’s disease and PCA relative to a healthy older control group in order to assess both the nature and the syndromic specificity of any auditory spatial deficits; neuroanatomical associations were assessed using voxel-based morphometry of patients’ brain magnetic resonance images. We hypothesized that the typical Alzheimer’s disease and PCA groups would show qualitatively similar deficits of auditory spatial analysis, but these deficits would be more severe in the PCA group given the neuroanatomical emphasis of this syndrome. We further hypothesized that auditory spatial impairment in these Alzheimer’s disease syndromes would correlate with grey matter atrophy in posterior temporo-parietal regions (posterior superior temporal lobe, temporo-parietal junction and precuneus) previously implicated in auditory scene analysis (Goll *et al.*, 2012).

## Materials and methods

### Participants

Twenty consecutive patients (seven female) fulfilling clinical criteria for typical Alzheimer’s disease with predominant episodic memory loss and additional cognitive dysfunction (Dubois *et al.*, 2007) and 12 patients (seven female) fulfilling criteria for PCA with predominant visual perceptual deficits and relatively preserved episodic memory (Tang-Wai *et al.*, 2004; Crutch *et al.*, 2012) participated. Syndromic diagnoses in the typical Alzheimer’s disease and PCA groups were corroborated with a comprehensive general neuropsychological assessment (Table 1). Brain MRI scans were available for review for 17 patients in the typical Alzheimer’s disease group and all patients in the PCA group: in the typical Alzheimer’s disease group, 12 patients showed a profile of disproportionate hippocampal volume loss with additional more widespread cortical atrophy and five patients showed diffuse cerebral atrophy; whereas in the PCA group, seven patients showed atrophy focused in posterior cortical areas with symmetrical involvement of the cerebral hemispheres and relative sparing of the hippocampi, four patients showed both posterior cortical and hippocampal atrophy and one patient showed mild generalized atrophy. No brain magnetic resonance images showed a significant cerebrovascular burden. Lumbar punctures and ^18^F-amyloid (Florbetapir) PET imaging (performed as part of another study) in 11 patients with typical Alzheimer’s disease and six patients with PCA showed a total CSF tau: amyloid-β_1-42_ ratio >1 or positive amyloid on visual rating of brain scans, compatible with underlying Alzheimer’s disease pathology in all cases. At the time of testing, in the typical Alzheimer’s disease group 17 patients were receiving symptomatic treatment with donepezil and one memantine; in the PCA group, 10 patients were receiving donepezil and two memantine. Twenty-six healthy age matched individuals (13 female) with no history of neurological or psychiatric illness also participated. No participant had a clinical history of hearing loss.

**Table 1:** General demographic, clinical and neuropsychological data for participant groups

Characteristics	Healthy controls^a^	Typical Alzheimer’s disease	PCA
**General**			
*n* (m:f)	26 (13:13)	20 (13:7)	12 (5:7)
Age (years)	66.7 (7.2)	66.0 (6.0)	60.5 (5.4)**
Education (years)	16.6(1.9)	14.3 (2.8)*	14.5 (1.7)*
MMSE (/30)	29.5 (1.0)	20.8 (4.5)*	20.2 (5.0)*
Symptom duration (years)	n/a	6.0 (2.7)	6.1 (3.2)
Symptomatic treatment (*n*)^b^	n/a	18	12
**Neuropsychological assessment**			
**Episodic memory**			
RMT Faces^†^ (Z-score)	0.24 (1.47)	−2.05 (1.72)*	−1.75 (2.4)*
RMT Words^†^ (Z-score)	0.89 (0.52)	−2.43 (1.07)*	−1.78 (2.19)*
**Executive skills**			
WASI Matrices (/32)^c^	24.4 (3.7)	12.1 (8.1)*	4.6 (5.0)**
WASI Block design (/71)	45.6 (18.0)	13.5 (12.4)*	–
WMS-R digit span forward (/12)^d^	9.2 (1.6)	6.8 (2.0)*	6.3 (2.1)*
WMS-R digit span reverse (/12)^d^	6.9 (2.0)	5.3 (2.6)*	3.3 (2.4)**
WMS-III spatial span forward (/16)^d^	7.3 (2.1)	5.4 (2.2)*	–
WMS-III spatial span reverse (/16)^d^	7.0 (1.7)	4.0 (2.2)*	–
**Verbal skills**			
WASI Vocabulary (/80)	70.0 (5.6)	51.3 (14.7)*	57.0 (9.0)*
WASI similarities (/48)	43.0 (8.0)	28.2 (8.8)*	–
GNT^††^ (/30)	26.5 (2.9)	15.4 (8.4)*	14.9 (6.5)*
BPVS (/150)	152.5 (22.6)	132.9 (22.9)*	–
NART (/50)^e^	44.0 (3.8)	32.6 (11.4)*	–
Schonell (/100)^f^	–	–	90.9 (5.8)*
**Posterior cortical skills**			
GDA (/24)^g^	14.4 (5.1)	6.3 (5.1)*	2.0 (3.0)**
VOSP Object Decision (/20)^h^	18.0 (2.2)	14.7 (2.4)*	9.5 (4.8)**
VOSP Dot Counting (/10)^d^	9.9 (0.3)	8.6 (2.6)*	3.6 (4.3)**

Maximum scores on neuropsychological tests (in parentheses) and mean (standard deviation) performance scores are shown unless otherwise indicated; results in bold indicate mean score <5th percentile; *significantly different from control group; **significantly different from control and other patient group (*P* < 0.05).

^†^PCA patients completed short Recognition Memory Test (25 items), typical Alzheimer’s disease patients completed long Recognition Memory Test (50 items), groups therefore not compared on this test.

^††^PCA patients completed Graded Naming Test to verbal definition.

– not administered.

Due to time constraints, subsets of participants completed particular tasks as follows.

^a^Data for 20 healthy controls unless otherwise stated; ^b^Donepezil or memantine (see text for details); ^c^10 PCA patients; ^d^26 healthy controls; ^e^19 typical Alzheimer’s disease patients; ^f^Nine PCA patients; ^g^18 typical Alzheimer’s disease patients; ^h^11 PCA patients.

BPVS = British Picture Vocabulary Scale (Dunn *et al.*, 1982); GDA = Graded Difficulty Arithmetic (Jackson and Warrington, 1986); GNT = Graded Naming Test (McKenna and Warrington, 1983); MMSE = Mini-Mental State Examination (Folstein *et al.*, 1975); NART = National Adult Reading Test (Nelson, 1982); RMT = Recognition Memory Test (Warrington, 1984) short Recognition Memory Test subtest of the Camden Memory Tests (Warrington, 1996); VOSP = Visual Object and Spatial Perception Battery (Warrington and James, 1991); WASI = Wechsler Abbreviated Scale of Intelligence (Wechsler, 1999); WMS-R = Wechsler Memory Scale-Revised (Wechsler, 1987); WMS-III = Wechsler Memory Scale 3rd edition (Wechsler, 1997).

Demographic and clinical details of the experimental groups are summarized in Table 1. All participants gave informed consent in accordance with the Declaration of Helsinki.

### Assessment of peripheral hearing

Peripheral hearing ability was assessed using pure tone audiometry, administered via headphones from a notebook computer in a quiet room. The procedure was adapted from a commercial screening audiometry software package (AUDIO-CDTM®, http://www.digital-recordings.com/audiocd/audio.html). Five frequency levels (500, 1000, 2000, 3000, 4000 Hz) were assessed: at each frequency, participants were presented with a continuous tone that slowly and linearly increased in intensity. Participants were instructed to indicate as soon as they were sure they could detect the tone; this response time was measured and stored for offline analysis. Hearing was assessed in each ear in each participant.

### Assessment of auditory spatial processing

#### General structure of the experimental battery

**Figure 1:**
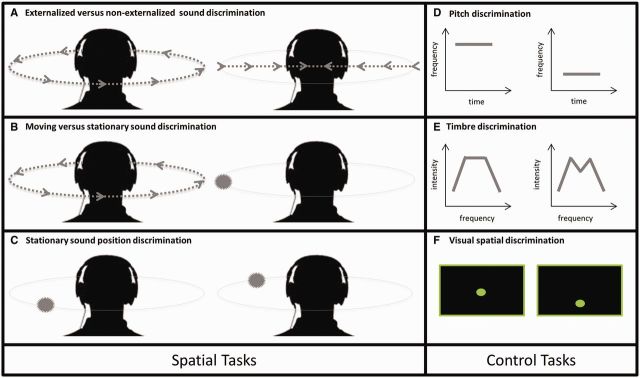
**Schematic representation of experimental battery**. Auditory spatial tasks are presented in the *left* hand panels (**A**–**C**); control tasks are presented in the *right* hand panels (**D**–**F**). Within each test panel, a stimulus pair corresponding to an experimental trial is shown; on a given trial, paired stimuli were presented sequentially with an intervening 1-s gap. In auditory spatial tests, perceived stimulus locations externalized in the azimuthal plane are shown; arrowed lines represent perceived trajectories of sound motion and filled circles represent perceived locations of stationary sounds. See text for details.

The experimental battery is schematized in Fig. 1; auditory stimulus characteristics are summarized in Table 2, further methodological details and sound examples are in the online Supplementary material. Sound sources in virtual acoustic space that were perceived either to remain stationary or revolve around the head were created digitally in Matlab®v7.0 by convolving a stereo broadband noise carrier with generic head-related transfer functions (HRTFs: Wightman and Kissler, 1989*a*, *b*). Convolution with HRTFs simulates the pinna filter functions and in normal listeners generates a percept of a sound source associated with a particular position in external space; sequential dynamic updating of HRTFs across different spatial positions simulates the perceptual effect of a moving sound source (Warren *et al.*, 2002). Five HRTF-specific versions of the externalized spatial stimulus set were created, allowing the corresponding generic HRTF to be matched with an individual participant’s gender and height (Supplementary Table 1 and Supplementary material). The carrier for all experimental auditory stimuli was iterated ripple noise (Yost, 1996): this carrier was chosen because it can be manipulated to code pitch variations as well as allowing convolution with HRTFs and was therefore suitable for constructing uniform auditory control as well as auditory spatial tasks (Warren and Griffiths, 2003).

**Table 2:** Summary of experimental auditory stimuli

Discrimination test	*n* trials	Condition	Duration sound^a^ (s)	Perceptual parameter	Magnitude parameter^b^	Start position in azimuth (deg)	Direction	Key manipulation	Spatial percept noise source
**Spatial tasks**										
Externalized versus non-externalized	20	Externalized^c^	3.2	Angular velocity	3.93 (rad/s)	90 or −90	Clockwise or anticlockwise	Dynamic update/ interpolate HRTFs	Revolving smoothly around head
		Non-externalized^d^		Interaural AM (beat)	1.25 (Hz)		‘Between’ ears	Composite HRTF	Swaying ‘through’ head
									
Moving versus stationary	60	Moving^c^	3.2	Angular velocity	0.33, 1.97 or 3.93 (rad/s)^bb^	45: 45: 315^f^	Clockwise or anticlockwise	Dynamic update / interpolate HRTFs	Revolving smoothly around head
		Stationary^e^		Static position	n/a		n/a	Positional HRTF, AM^g^	Stationary in space, with *vibrato*
									
Stationary sound position	60	Same / different location^e^	1	Step in azimuth	0, 30, 45 or 60 (deg) ^bb^	30: 30: 330^ff^	Clockwise or anticlockwise inter-sound ‘step’	Positional HRTF	Stationary in space
**Control tasks**										
Pitch	20	Same / different pitch	1	Frequency step	0 or 8.3 (Hz)^bbb^	n/a	Higher or lower	n/a	n/a
									
Timbre^h^	40	Same / different spectral shape	1	Envelope amplitude attenuation	0, 10 or 50 (%)	n/a	n/a	n/a	n/a

Stimulus characteristics and associated spatial percepts and the number of trials for each test (maximum scores) are shown. All tests were based on a two-alternative-forced-choice (‘same / different?’) decision; the proportion of ‘same’ and ‘different’ trials was equal in each test. Where sound pairs included externalized stimuli, sounds in each pair were matched for start position and direction.

^a^Duration of individual sounds in a trial pair; ^b^Level of key perceptual parameter; ^bb^Level or ‘difficulty’ blocks; ^bbb^Non-musical pitch intervals used to reduce reliance on past musical experience; ^c^Adapted from previously described method (Warren *et al.*, 2002); ^d^Adapted from previously described method (Joris *et al.*, 2006); ^e^Adapted from previously described method (Warren and Griffiths, 2003); ^f^Sound starting position roved across trials between 45° and 315° in azimuth, in 45° steps between trials; ^ff^Sound starting position roved across trials between 30° and 330° in azimuth, in 30° steps between trials; ^g^Amplitude modulation applied binaurally to stationary sounds at equivalent rates to angular velocities of moving sounds, to control for overall dynamic flux; ^h^Adapted from previously described method (Goll *et al.*, 2010).

AM = amplitude modulation; deg = degrees; n/a = not applicable; rad = radians. Further details of experimental methods can be found in the Supplementary material.

We created novel tests to probe three dimensions of auditory spatial analysis: discrimination of sounds localized in external space from non-externalized sounds (perceived as originating between the ears, as when listening for example to a personal sound system via headphones); discrimination of moving from stationary externally localized sounds; and discrimination of stationary sounds at different locations in external space. These dimensions of auditory spatial analysis are relevant for processing real auditory environments and have been shown to engage dorsal auditory cortical pathways (Clarke *et al.*, 2002; Warren *et al.*, 2002; Zatorre *et al.*, 2002; Smith *et al.*, 2010). To minimize extraneous cognitive demands from cross-modal labelling and executive processes that are potentially vulnerable in Alzheimer’s disease (Stopford *et al.*, 2012), all experimental tests were based on a uniform two-alternative-forced-choice (‘1-back’) response procedure requiring the participant to make ‘same/different?’ judgements on pairs of sounds presented serially. Sound durations were fixed within an experimental test and the sounds in each pair were separated by a 1-s silent gap; sound level was roved over experimental trials but fixed for a given trial. Where feasible, the key experimental perceptual parameter in a test was manipulated to create different parameter ‘difficulty’ levels, to allow us to assess a wider range of auditory spatial competence in patients and healthy individuals (Table 2 and Supplementary material).

Auditory control tasks based on timbre and pitch discrimination with other parameters matched to the spatial tests were designed to index spectrotemporal processing and non-verbal auditory working memory, respectively. Finally, to compare auditory and visual spatial processing in the typical Alzheimer’s disease group, participants were assessed on tests of visual spatial processing and visual motion perception; only the visual motion task was administered to patients in the PCA group.

#### Externalized versus non-externalized sound discrimination

The key factor assessed in this test was perception of cues relevant to any external location of a sound: we created conditions to compare sounds matched for dynamic properties (Joris *et al.*, 2006) where the only parameter manipulated was the externalizing effect of the HRTF, such that sounds were perceived as either externalized or non-externalized (Table 2 and Supplementary material).

#### Moving versus stationary sound discrimination

For this test, we used HRTF filtering to create moving sounds perceived as revolving externally around the head with constant angular velocity (one of three values, varied between trials), and these moving sounds were compared with stationary sounds perceived as located in external space (at different locations between trials), by convolving with HRTFs (Table 2 and Supplementary material). Amplitude modulation was applied binaurally to stationary sounds to match overall spectrotemporal variation between moving and stationary conditions.

#### Stationary sound position discrimination

For this test, pairs of sounds normally perceived as stationary in external space were created by convolving with HRTFs corresponding to pairs of positions around the head; sound positions in a pair were either the same (‘same’ trials) or separated by a spatial step (one of three values, varied between trials; ‘different’ trials, Table 2 and Supplementary material).

#### Auditory control tests

To create a control test to assess complex spectrotemporal (timbre) discrimination, the spectral shape of the noise carrier (the set of relative intensity weightings of the individual frequencies composing the noise) was manipulated using a previously described method (Goll *et al.*, 2010) to create two levels of task difficulty. In addition, a control pitch discrimination test to assess non-verbal auditory working memory was created by varying pitch of the noise carrier (Table 2, Fig. 1 and Supplementary material).

#### Experimental procedure for auditory tests

For tests in which the level of perceptual parameters was varied, trials at a given level were administered as blocks (each comprising 20 trials), to allow testing to be discontinued if a participant’s performance fell to chance (in which case a chance score was attributed for the next block). For a given test or block, paired sounds were either the same (10 trials) or different (10 trials) according to the parameter of interest, presented in randomized trial order. Sounds were delivered as digital wavefiles via headphones at a comfortable listening level (at least 70 dB) and responses were collected for off-line analysis using a notebook computer running Matlab®v7.0 and the Cogent v1.25 extension. The task on each trial was to decide if the two sounds were the same or different. No feedback about performance was given and no time limits were imposed. Before testing, participants were familiarized with the experimental procedures, including practice trials; visual aids were used where possible, to ensure the participant understood the task (Supplementary Fig. 1 and Supplementary material).

#### Visual spatial tests

The novel control test to assess visual spatial perception was analogous to the auditory stationary position discrimination test and required participants to discriminate the spatial positions of sequentially presented, paired circles using a two-alternative-(same/different)-forced-choice response procedure (details in the Supplementary material); the test comprised 60 trials (three blocks of 20 trials at different difficulty levels). This test was administered to the typical Alzheimer’s disease and healthy control groups but not the PCA group. A subset of participants from all three groups (14 healthy controls, 13 typical Alzheimer’s disease, 11 PCA) completed a further test of visual motion coherence perception on dot arrays (adapted from Braddick *et al.*, 2000) in which the task on each trial was to decide whether coherent motion was present (details in Supplementary material); this test comprised 80 trials (four blocks of 20 trials at different difficulty levels).

### Behavioural analyses

Demographic data on age, education, Mini-Mental State Examination score, and symptom duration were analysed using linear regression models; a Chi-square test of distribution was used to assess whether gender distribution differed significantly between experimental groups. As experimental data did not conform to normality assumptions, we implemented a cluster-adjusted logistic regression model with robust standard error to assess odds of correct response (odds ratio, OR), with auditory spatial task types (discrimination of externalized versus non-externalized sounds, moving versus stationary sounds, stationary sound position), auditory control and visual task types (timbre, pitch, visual spatial and motion coherence) and group (healthy control, typical Alzheimer’s disease, PCA) entered concurrently as predictors of interest. Interactions between group and test type were fitted to assess group-associated effects on particular tasks whilst controlling for performance on other tasks. Age, peripheral hearing performance (see also Supplementary material on-line), years in education and reverse digit span (as an index of both auditory working memory capacity and disease severity: Baddeley *et al.*, 1991; Perry and Hodges, 1999) were included as additional covariates of no interest. The Wald criterion was used to determine specific effects of patient group on total correct response in each experimental task. Correlations between experimental task scores and neuropsychological variables were assessed using Spearman’s rank tests. We also examined further those tests that included blocks of varying perceptual parameter level, using d-prime as an index of discriminability. We used linear regression models with robust standard error to assess the effect of perceptual parameter level on discriminability for each task type and experimental group separately, controlling for age and peripheral hearing performance.

### Brain image processing

#### Image acquisition

At the time of behavioural assessment, 17 patients in the typical Alzheimer’s disease group and all patients in the PCA group underwent volumetric brain MRI on a Siemens 3 T Trio scanner using a 32-channel phased array head coil. T_1_-weighted volumetric images were obtained using a sagittal 3D magnetization prepared rapid gradient echo sequence (echo time/repetition time/inversion time = 2.9/2200/900 ms, dimensions of 256 × 256 × 208, voxel size of 1.1 × 1.1 × 1.1 mm).

#### Voxel-based morphometry

Preprocessing of patient brain magnetic resonance images for voxel-based morphometry was performed using New Segment and the DARTEL toolbox of SPM8 (www.fil.ion.ucl.ac.uk/spm) running under Matlab2012a® (Ashburner, 2007; Ridgway *et al.*, 2008). Normalization, segmentation and modulation of grey and white matter images were performed using default parameter settings, with a smoothing Gaussian full-width at half-maximum of 6 mm. To adjust for individual differences in global grey matter volume during subsequent analysis, total intracranial volume was calculated for each participant by summing grey matter, white matter and CSF volumes following segmentation of all three tissue classes. A study-specific mean brain image template was created by warping all bias-corrected native space whole-brain images to the final DARTEL template and calculating the average of the warped brain images.

Generalized linear models were used to examine regional grey matter volume correlations with performance on auditory experimental tasks for which the combined patient cohort exhibited deficits compared to the healthy control group in the behavioural analysis. For each task, voxel intensity (grey matter volume) was modelled as a function of experimental test score across the combined patient cohort, within each syndromic group and comparing syndromic groups, including syndromic group, age, total intracranial volume, gender and reverse digit span total score as covariates of no interest. In addition, grey matter correlates of performance on the visual spatial discrimination tasks within the typical Alzheimer’s disease group was assessed in a separate model. To help protect against voxel drop-out due to marked local regional atrophy, we applied a customized explicit brain mask based on a specified ‘consensus’ voxel threshold intensity criterion (Ridgway *et al.*, 2009) whereby a voxel was included in the analysis if grey matter intensity at that voxel was > 0.1 in > 70% of participants (rather than in all participants, as with the default SPM8 mask).

Statistical parametric maps (SPMs) of regional grey matter volume correlating with score on each auditory experimental test were examined at threshold *P* < 0.05 after family-wise error (FWE) correction for multiple comparisons over the whole brain and after small volume correction using anatomical regions based on our previous anatomical hypotheses. Anatomical small volumes were derived from the Oxford-Harvard brain maps (Desikan *et al.*, 2006) in FSLview (Jenkinson *et al.*, 2012) and edited using MRIcron (www.mccausandcentre.sc.edu/micro/micron) for the study-customized template brain image. These small volumes included key areas previously implicated in auditory scene analysis and spatial processing (Warren *et al.*, 2002; Zatorre *et al.*, 2002; Warren and Griffiths, 2003; Arnott *et al.*, 2004; Spierer *et al.*, 2008; Goll *et al.*, 2012): posterior superior temporal lobe and inferior parietal lobe (supramarginal and angular gyri) and retrosplenial cortex (posterior cingulate and precuneus) in each cerebral hemisphere.

## Results

### General characteristics

Participant groups did not differ significantly in gender distribution and patient groups did not differ on global measures of disease stage and severity (Mini-Mental State Examination score, symptom duration; Table 1). Whereas the typical Alzheimer’s disease and healthy control groups were well matched for age, the PCA group was on average significantly younger than both the control group [beta = 6.18, 95% confidence interval (CI) 4.5 to 7.8, *P* < 0.001] and the typical Alzheimer’s disease group [beta = 5.67, CI 3.9 to 7.4, *P* < 0.001]. Both the typical Alzheimer’s disease group [beta = −2.37, CI −2.8 to −1.9, *P* < 0.001] and PCA group [beta = −2.10, CI −2.7 to −1.5, *P* < 0.001] had significantly fewer years of education than the healthy control group. Both patient groups showed the anticipated syndromic neuropsychological profiles (Table 1): the typical Alzheimer’s disease group showed marked impairment of episodic memory with additional more widespread cognitive deficits relative to the healthy control group, whereas the PCA group showed marked deficits of visual spatial perception, arithmetic and non-verbal reasoning with less severe episodic memory impairment than the typical Alzheimer’s disease group. Group membership had no significant effect on audiometry performance (details in Supplementary material); however, peripheral hearing performance was included as a covariate in further analyses to account for any confounding effect of this factor.

### Experimental task performance

#### Auditory spatial tasks

**Figure 2:**
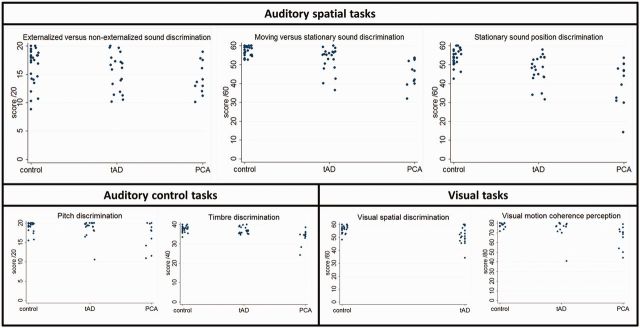
**Raw data.** Individual raw data are plotted for each experimental test for the healthy control group (control), the patient group with a typical syndrome of Alzheimer’s disease (tAD) and the patient group with a syndrome of posterior cortical atrophy (PCA).

A summary of experimental test performance for each group is presented in Table 3; individual data are in Fig. 2 (see also Supplementary material). Qualitatively, healthy control participants and patients all perceived the effect of HRTF convolution as a sound source in virtual acoustic space. The healthy control group performed at sub-ceiling level on experimental tests apart from externalized versus non-externalized sound discrimination, for which control performance was more variable. There was a significant interaction between patient group and test type [χ^2^(11) = 28.6, *P* = 0.003]. Both the typical Alzheimer’s disease group and the PCA group performed comparably to healthy controls on externalized versus non-externalized sound discrimination [typical Alzheimer’s disease: OR = 0.87, CI 0.5 to 1.6, *P* = 0.64; PCA: OR = 0.74, 95% CI 0.4 to 1.5, *P* = 0.40]. However, both patient groups performed significantly worse than controls on both moving versus stationary sound discrimination [typical Alzheimer’s disease: OR = 0.36, CI 0.2 to 0.7, *P* = 0.001; PCA: OR = 0.20, CI 0.1 to 0.4, *P* < 0.001] and stationary sound position discrimination [typical Alzheimer’s disease: OR = 0.46, CI 0.3 to 0.7, *P* = 0.001; PCA: OR = 0.31, CI 0.2 to 0.6, *P* < 0.001]. The PCA group performed significantly worse than the typical Alzheimer’s disease group on moving versus stationary sound discrimination [OR = 0.55, CI 0.3 to 0.9, *P* = 0.03] but there were no significant performance differences between the patient groups on stationary sound position discrimination [OR = 0.67, CI 0.4 to 1.2, *P* = 0.18].

**Table 3:** Summary of group performance on experimental tasks

Task	Max. score	Healthy controls	Typical Alzheimer’s disease	PCA
**Auditory spatial discrimination**				
Externalized versus non-externalized sounds	20^a^	16.5 (3.2)	15.3 (3.4)	14.1 (3.2)
Moving versus stationary sounds	60	57.6 (2.3)	52.2 (6.5)*	45.4 (6.7)**
Stationary sound position	60	54.3 (4.0)	46.7 (7.7)*	39.9 (11.8)*
**Auditory control**				
Pitch discrimination	20	19.2 (1.3)	18.6 (2.3)	17.3 (3.5)
Timbre discrimination	40^b^	37.9 (1.5)	36.6 (1.6)	33.3 (4.0)**
**Visual spatial**				
Spatial discrimination	60^c^	57.0 (2.8)	50.8 (5.9)*	n/a
Motion coherence perception	80^d^	78.4 (2.4)	73.9 (10.1)*	65.5 (12.2)*

Group raw scores on auditory and visual experimental tasks are shown; mean (SD) values are presented (individual data are plotted in Fig. 2).

*Significantly different from control group; **Significantly different from control and other patient group (*P* < 0.05). Because of time constraints, subsets of participants completed particular tasks as follows.

^a^Nineteen patients with typical Alzheimer’s disease, 11 patients with PCA.

^b^Nineteen patients with typical Alzheimer’s disease.

^c^Eighteen patients with typical Alzheimer’s disease.

^d^Fourteen healthy controls, 13 patients with typical Alzheimer’s disease, 11 patients with PCA.

#### Auditory and visual control tasks

On both auditory control tasks, the healthy control and typical Alzheimer’s disease groups performed comparably [pitch discrimination: OR = 0.65, CI 0.3 to 1.7, *P* = 0.38; timbre discrimination: OR = 0.78, CI 0.5 to 1.2, *P* = 0.25]; whereas the PCA group showed a trend towards inferior pitch discrimination performance relative to healthy controls [OR = 0.38, CI 0.1 to 1.1, *P* = 0.07] and a deficit of timbre discrimination relative both to healthy controls [OR = 0.41, CI 0.2 to 0.7, *P* = 0.003] and the typical Alzheimer’s disease group [OR = 0.53, CI 0.3 to 0.8, *P* = 0.004]. On experimental tests of visual spatial function, relative to the healthy control group the typical Alzheimer’s disease group showed impaired visual spatial discrimination [OR = 0.37, CI 0.2 to 0.7, *P* = 0.001] (the PCA group was not assessed on this task due to the severity of visual spatial impairment in this group; Table 1) and both patient groups showed impaired visual motion coherence perception [typical Alzheimer’s disease: OR = 0.33, CI 0.1 to 1.0, *P* = 0.049; PCA: 0.15, CI 0.05 to 0.4, *P* < 0.001]; there were no differences between the typical Alzheimer’s disease and PCA groups [OR = 0.44, CI 0.1 to 1.4, *P* = 0.16].

#### Correlation analyses

Correlations between experimental task performance and general neuropsychological functions are summarized in Supplementary Table 2. Performance on experimental tests in the patient groups was significantly positively correlated with a standard measure of general cognitive severity (Mini-Mental State Examination score). There was also a significant positive correlation with pitch and moving versus stationary sound discrimination for both patient groups, and between pitch and sound position discrimination in the typical Alzheimer’s disease group only. Visual spatial discrimination performance correlated with moving versus stationary sound, sound position and pitch discrimination in the typical Alzheimer’s disease group. Performance on the visual motion coherence task correlated with moving versus stationary sound discrimination for both patient groups.

#### Perceptual parameter analysis

**Figure 3:**
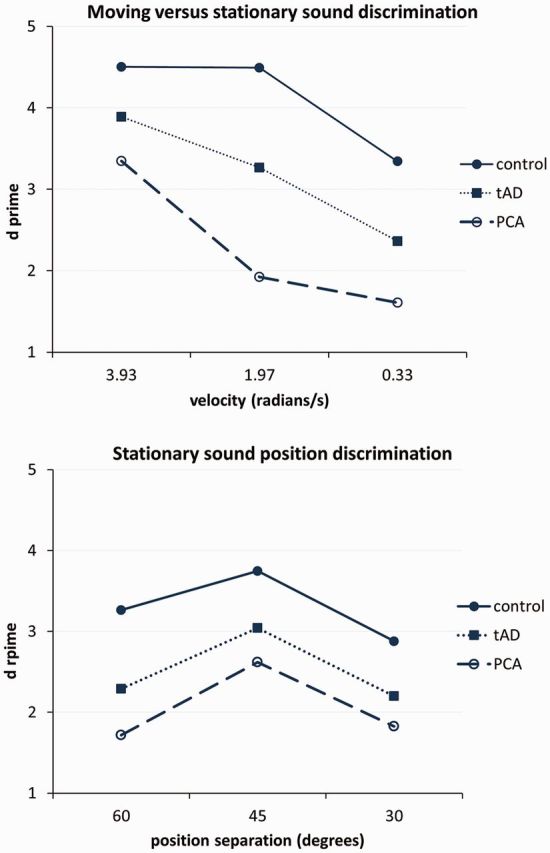
**Discriminability by parameter level.** Mean d-prime scores are plotted for each perceptual parameter level/condition for the moving versus stationary and stationary sound position discrimination tasks. Unbroken lines represent healthy controls; dotted lines the patient group with a typical syndrome of Alzheimer’s disease (tAD); and dashed lines the patient group with a syndrome of posterior cortical atrophy (PCA).

Across groups, performance on the moving versus stationary sound discrimination and timbre discrimination tests was correlated with the prescribed task difficulty level (magnitude of the relevant stimulus parameter); whereas performance on the stationary sound position discrimination test was not monotonically related to perceptual parameter level but rather showed a falling off of discriminability at the largest spatial separation (Fig. 3; d-prime values in Supplementary Table 3).

### Neuroanatomical associations

**Figure 4:**
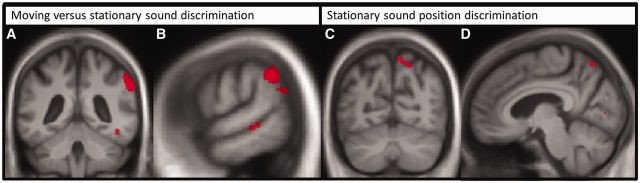
**Brain imaging.** Statistical parametric maps of associations of regional grey matter volume with performance on experimental auditory spatial tasks in the combined patient group. Maps are thresholded at an uncorrected whole-brain significance level *P* < 0.001 for display purposes. Maps are projected on coronal (**A** and **C**), and sagittal (**B** and **D**) sections of the mean patient cohort T_1_-weighted brain MRI; the right hemisphere is shown on the right in coronal sections. Grey matter associations of moving versus stationary sound discrimination (**A** and **B**) and stationary sound position discrimination (**C** and **D**) are indicated.

In the neuroanatomical analysis, grey matter associations of performance on moving versus stationary sound discrimination and stationary sound position discrimination were assessed as these tasks showed disease-associated behavioural deficits (Fig. 4; further details in Supplementary Table 4 and Supplementary Fig. 2). In the combined patient cohort, performance on the moving versus stationary sound discrimination task was positively correlated with grey matter volume in right inferior parietal lobe [peak Montreal Neurological Institute (MNI) stereotactic space coordinates (62 −45 36)], thresholded at *P* < 0.05 after FWE correction for multiple comparisons over the whole brain. No additional grey matter associations of moving versus stationary sound discrimination were identified at the prescribed threshold after correction within the small volumes of interest specified by our prior anatomical hypotheses; however, at a more lenient uncorrected threshold (*P* < 0.001 over the whole brain volume), additional cerebral correlates of moving versus stationary sound discrimination were identified in left temporo-parieto-occipital junction, right posterior superior temporal sulcus, right fusiform gyrus and basal ganglia (Supplementary Table 4). Performance on the stationary sound position discrimination task for the combined patient cohort was positively correlated with grey matter volume in right precuneus [peak MNI coordinates (8 −66 58)], thresholded at *P* < 0.05 after FWE correction for multiple comparisons within the small volume of interest specified by our previous anatomical hypotheses. No grey matter regions showing a significant inverse association with auditory spatial task performance were identified.

Assessed separately, the typical Alzheimer’s disease and PCA groups showed no significant grey matter associations of performance on either spatial task at the prescribed threshold nor were any significant intergroup differences in regional grey matter associations of auditory spatial performance identified at this corrected threshold. Visual spatial discrimination performance within the typical Alzheimer’s disease group had a positive grey matter correlate in right precuneus near that identified for auditory spatial discrimination in the combined patient cohort [peak MNI coordinates [9 −76 45)], at a lenient uncorrected threshold (*P* < 0.001 over the whole brain volume).

## Discussion

Here we have shown that clinically typical amnestic Alzheimer’s disease and PCA (the major visual variant phenotype of Alzheimer’s disease) are both associated with impaired auditory spatial processing. The two syndromic groups showed broadly similar profiles of auditory spatial deficits relative to healthy older controls, with sparing of discriminability of cues to sound externalization but impaired discrimination of sound motion and static position in external space. Auditory spatial performance showed a similar relation to perceptual parameters and task difficulty in healthy controls and both patient groups (Fig. 3), suggesting that the tasks were accessing similar perceptual mechanisms across groups (the non-monotonic relation for stationary sound position discrimination may reflect confusion between sound locations in front and behind the head over large spatial steps, as described in previous psychophysical work: Middlebrooks and Green, 1991; Blauert, 1997). PCA was associated with significantly greater impairment of sound motion processing than typical Alzheimer’s disease. These auditory spatial deficits were accompanied by correlated deficits in the processing of visual spatial location and motion in both syndromic groups, in keeping with some convergence of mechanisms (or perhaps, multimodal processing) of spatial information in the auditory and visual domains; this was corroborated by qualified evidence for anatomical convergence between spatial modalities in the typical Alzheimer’s disease group here, though the lack of robust neuroanatomical associations within particular syndromic groups (likely in part reflecting the relatively small case numbers) suggest a need for caution in interpreting any convergence of brain mechanisms of spatial analysis based on behavioural findings. Although auditory spatial performance correlated with patients’ working memory capacity in both verbal and non-verbal domains, the auditory spatial deficits demonstrated were not attributable simply to this factor. With the caveat that power to detect weaker effects was relatively low, tests of non-spatial auditory processing appeared largely spared in the typical Alzheimer’s disease group, whereas the PCA group showed an impairment of complex spectrotemporal (timbre) processing and a trend toward deficient pitch discrimination. We do not therefore argue that central auditory dysfunction in these syndromes is restricted to auditory spatial processing: indeed, a broader profile of central auditory impairment has been documented previously in Alzheimer’s disease (Kurylo *et al.*, 1993; Strouse *et al.*, 1995; Gates *et al.*, 1996, 2008, 2011; Golob *et al.*, 2007, 2009; Goll *et al.*, 2011). Here, however, auditory spatial deficits in typical Alzheimer’s disease and PCA were demonstrated after accounting for non-spatial, complex auditory and peripheral hearing function. Taken together, our findings suggest that impaired coding of external space in Alzheimer’s disease extends beyond vision to the realm of sound; and further, that this impairment may be relatively selective for auditory spatial versus other kinds of complex auditory information. The findings corroborate previous evidence for impaired auditory scene analysis in Alzheimer’s disease (Goll *et al.*, 2012).

Structural neuroanatomical correlates of auditory spatial processing in the present patient cohort were identified in non-dominant parietal cortex. This neuroanatomical association accords with a substantial body of previous evidence from functional neuroimaging and neuropsychological studies in the healthy and damaged brain implicating parietal lobe areas in various aspects of auditory spatial analysis within the dorsal auditory cortical processing network (Clarke *et al.*, 2002; Lewald *et al.*, 2002; Arnott *et al.*, 2004; Brunetti *et al.*, 2005; Alain *et al.*, 2008). The parietal lobe has a well-established role in visual and multimodal spatial processing (Bushara *et al.*, 1999; Bremmer *et al.*, 2001; Rizzolatti and Matelli, 2003; Cohen, 2009) and is likely to be critical for the formation of an egocentric spatial reference frame across sensory modalities (Karnath, 1997; Bellmann *et al.*, 2001; Krumbholz *et al.*, 2005). Such a role would be in line with the requirements of the present experimental tasks, which demanded analysis of sounds referenced to (virtual) egocentric acoustic space. Any apparent hemispheric lateralization of correlates here should be interpreted with caution; of interest, however, the balance of previous evidence suggests that the right parietal lobe may instantiate more specialized mechanisms for auditory spatial analysis whereas the left hemisphere may play a more restricted role in auditory spatial processing (Clarke *et al.*, 2002; Zatorre and Penhune, 2001; Zimmer *et al.*, 2003; Arnott *et al.*, 2004; Krumbholz *et al.*, 2005). The additional cerebral correlates of auditory spatial processing identified here using a relaxed criterion (Supplementary Table 4) should be interpreted with caution. Nevertheless, these additional grey matter correlates are also in line with previous functional imaging work in the healthy brain implicating posterior superior temporal cortices in both hemispheres in the disambiguation of auditory spatial from object identity characteristics and obligatory cross-modal information processing (Zatorre *et al.*, 2002; Warren and Griffiths, 2003; Arnott *et al.*, 2004; Spierer *et al.*, 2008) and subcortical structures including the basal ganglia in auditory sequencing and tracking of auditory information (Arnott *et al.*, 2004; Pastor *et al.*, 2006).

Our findings further suggest that critical neuroanatomical substrates for processing sound motion and static sound location are separable. It remains unclear whether the cognitive mechanisms that process particular auditory spatial parameters can be differentiated (Middlebrooks and Green, 1991; Blauert, 1997; Ducommun *et al.*, 2002, 2004; Richter *et al.*, 2013); however, the present neuroanatomical data are in line with previous work in the healthy brain and in focal brain damage implicating temporo-parietal junction and precuneus in the analysis of sound motion and static location, respectively (Warren *et al.*, 2002; Ducommun *et al.*, 2004; Krumbholz *et al.*, 2005; Zündorf *et al.*, 2013). These correlates might in turn reflect the relative dependence of auditory motion coding on fine-grained spectrotemporal analysis and auditory location discrimination on imagery processes that integrate stored auditory representations (Griffiths and Warren, 2002; Warren *et al.*, 2002; Zündorf *et al.*, 2013; Zvyagintsev *et al.*, 2013). Involvement of precuneus here further accords with previous work implicating retrosplenial cortex in auditory scene analysis in Alzheimer’s disease (Goll *et al.*, 2012). Though caution is required in light of the convergence of behavioural deficits for processing auditory static and dynamic spatial cues in this neurodegenerative disease cohort, these separable neuroanatomical correlates are in line with previous evidence for a dedicated velocity detection mechanism underpinning perception of sound movement (Griffiths *et al.*, 1996; Carlile and Best, 2002).

The more severe impairment of auditory motion analysis and impaired timbre processing in the PCA group amplifies previous work suggesting that patients with PCA have particular difficulty tracking auditory information streams and with spectrotemporal feature processing (for example, in prosody) as well as with visual spatial analysis (Crutch *et al.*, 2013). Although the pathological substrates in the PCA cohort await individual substantiation, collective experience suggests that the great majority will have underlying Alzheimer’s disease pathology (Tang-Wai *et al.*, 2004; Crutch *et al.*, 2012). Taken together, the present behavioural and neuroanatomical findings suggest that the profile of auditory spatial impairment in Alzheimer’s disease is modulated to some degree by clinical phenotype. However, it is noteworthy that the neuroanatomical regions correlating with auditory spatial processing across the present patient cohort are core components of default mode network (Greicius *et al.*, 2009; Seeley *et al.*, 2009); moreover, these parietal cortical areas have been identified in previous work as sites of common involvement in Alzheimer’s disease variant syndromes (Lehmann *et al.*, 2011, 2013; Warren *et al.*, 2012). Auditory spatial analysis (and auditory scene analysis more generally) may be a sensitive probe of default mode network integrity, perhaps because it demands precise tracking of events over time and integration of incoming sensory data with internalized templates (Goll *et al.*, 2012; Zündorf *et al.*, 2013): an instance of self monitoring in relation to environment, proposed as a generic function of default mode network in other contexts (Buckner and Carroll, 2007; Buckner *et al.*, 2008). Although the disambiguation of externalized from non-externalized auditory percepts might also have been predicted *a priori* to index default mode network function, it is noteworthy that performance on this task showed relatively wide variability in our healthy control group, in line with previous evidence suggesting that additional factors (such as head movement) may operate under natural listening conditions (Brimijoin *et al.*, 2013). Taking all the present data into account, the processing of auditory spatial like certain other forms of sensory information (Witoonpanich *et al.*, 2013) may transcend conventional syndromic boundaries to index generic mechanisms that are damaged in common at least in posterior variant Alzheimer’s disease phenotypes.

From a clinical perspective, this study highlights a potential brain basis for a poorly characterized class of symptoms reported by patients with Alzheimer’s disease, with potential implications for environmental design and modification. Although frank auditory disorientation is described infrequently, difficulty interacting with complex auditory environments is commonly experienced by patients with Alzheimer’s disease and may be erroneously attributed to age related peripheral hearing impairment. Auditory dysfunction in Alzheimer’s disease may lead to social withdrawal and disability as well as compounding cognitive deficits (Petitot *et al.*, 2007; Dhanjal *et al.*, 2013). In addition, the correlation of auditory spatial performance with a global cognitive index in the Alzheimer’s disease cohort here suggests that aspects of auditory scene analysis may track disease and might potentially constitute a means of probing and tracking Alzheimer’s disease evolution across Alzheimer’s disease phenotypes. The present findings provide a clinical and neurobiological rationale for more systematic and detailed analysis of auditory spatial function in Alzheimer’s disease, with several avenues for future work. Auditory spatial functions should be assessed longitudinally alongside other central and peripheral auditory processes in larger patient cohorts with Alzheimer’s disease, in relation both to other neurodegenerative disease cohorts and comparing the major Alzheimer’s disease variant syndromes. In this regard, logopenic aphasia, which targets temporo-parietal cortex (Warren *et al.*, 2012) and produces various non-verbal auditory deficits (Goll *et al.*, 2011) may be particularly informative. The potential of auditory spatial processing to probe and elucidate brain network dysfunction across the Alzheimer’s disease spectrum warrants further investigation with both structural and functional neuroanatomical and ultimately, neuropathological substantiation.

## Abbreviations

HRTF: head-related transfer function
PCA: posterior cortical atrophy
